# Higher glomerular filtration rate is related to insulin resistance but not to obesity in a predominantly obese non-diabetic cohort

**DOI:** 10.1038/srep45522

**Published:** 2017-04-03

**Authors:** Negar Naderpoor, Jasmine G. Lyons, Aya Mousa, Sanjeeva Ranasinha, Maximilian P. J. de Courten, Georgia Soldatos, Barbora de Courten

**Affiliations:** 1 Monash Centre for Health Research and Implementation, School of Public health and Preventive Medicine, Monash University, Melbourne, Australia; 2 Diabetes and Vascular Medicine Unit, Monash Health, Melbourne, Australia; 3 School of Population and Global Health, University of Melbourne, Melbourne, Australia; 4 Centre for Chronic Disease Prevention and Management, Victoria University, Melbourne, Australia

## Abstract

Glomerular hyperfiltration has been associated with obesity, insulin resistance, and systolic blood pressure (SBP). However, previous studies are limited by confounders such as pre-existing diabetes or hypertension, or have used indirect measures of adiposity and insulin sensitivity (IS). Therefore, we examined the relationship between estimated glomerular filtration rate (eGFR) and IS measured by the hyperinsulinaemic euglycaemic clamp in a healthy population on no medications. We performed oral glucose tolerance test (OGTT) and measured % body fat (DEXA), BMI, blood pressure and M-value (hyperinsulinaemic euglycaemic clamp) in 104 individuals (44 females and 60 males). The majority of the study population (n = 89, 85.6%) were classified on their BMI as overweight/obese. eGFR was related to age, BMI, M-value (IS), 2-hour glucose levels post OGTT and white blood cell count (WBC) (all p < 0.05); but not to SBP (p = 0.1) or fasting glucose levels (p = 0.2). After adjustment for gender, BMI, SBP and WBC, the inverse association between eGFR and M-value (p = 0.001), and 2-hour glucose post OGTT (p = 0.02) persisted. In conclusion, although eGFR has been associated with BMI and blood pressure in previous studies, in our healthy population, eGFR was more closely related to markers of glucose metabolism (IS and 2-hour glucose post OGTT) than to BMI and blood pressure.

Glomerular hyperfiltration, which is an abnormally high glomerular filtration rate (GFR), has been reported to predict incident diabetes and progression to chronic kidney disease^1^,^2^,^3^. It is estimated to occur in up to 70% of patients with type 1 and 40% of those with type 2 diabetes at the time of diagnosis or in the early stages of disease^4^. Glomerular hyperfiltration is associated with increased risk of albuminuria and faster progression to diabetic nephropathy in patients with diabetes^1^,^4^,^5^. Obesity and insulin resistance, two key risk factors for type 2 diabetes, have also been associated with hyperfiltration^3^,^6^,^7^,^8^,^9^. In addition, hypertension, which is common among people with obesity and insulin resistance, also increases hyperfiltration^1^. Therefore, it is not surprising that the presence of glomerular hyperfiltration has been identified prior to the diagnosis of overt type 2 diabetes^2^,^10^. In a large community-based population, the prevalence of hyperfiltration increased with increasing fasting plasma glucose levels among those with prediabetes^2^. The mechanisms underlying hyperfiltration are not fully understood; however key processes responsible for altering renal haemodynamics associated with hyperfiltration relate to the imbalance of afferent/efferent arteriolar resistance and increased renal blood flow^1^. Importantly, amelioration of glomerular hyperfiltration in type 2 diabetes has been shown to be associated with both improved insulin sensitivity (IS) and a slower decline in renal function over time^5^. Gender differences have been reported in factors affecting insulin resistance as well as renal function and progression to chronic kidney disease^11^,^12^,^13^,^14^. Sex hormones, body composition, and fat distribution are possible underlying mechanisms^12^,^14^.

To date, observational studies investigating associations between GFR and IS have been very heterogeneous in both study population and methodology. They often include patients with comorbid conditions such as obesity, diabetes, hypertension, and different GFR categories, and as such, are unable to separate completely the effect of these confounders on renal function^15^. Furthermore, methods of measuring renal function and IS vary between studies and are often not the gold standard. Hence, results may be affected by confounders or may not be generalizable to the wider population. Our study aim was to investigate the relationship between eGFR calculated by the CKD-EPI (Chronic Kidney Disease Epidemiology Collaboration) formula^16^, adiposity, and IS measured by the gold standard hyperinsulinaemic euglycaemic clamp^17^ in non-diabetic, normotensive, and drug naive individuals. Multivariable linear regression models were used to assess these relationships, and to determine other significant predictors of eGFR. We also examined gender differences in factors associated with eGFR.

## Methods

### Participants

Our sample consisted of 104 predominantly obese but otherwise healthy volunteers (44 female and 60 male) from the community in Melbourne, aged 18 to 57 years (median ± interquartile range 29 ± 14) who underwent a rigorous protocol employing measures of obesity, adiposity, and insulin sensitivity. Participants were part of our ongoing studies investigating risk factors for cardiometabolic diseases in healthy individuals. Being overweight/obese based on BMI was an inclusion criterion for one of these studies. Phone screening using a standard questionnaire was conducted initially for each volunteer to ensure that they did not have any co-morbidities, did not take any medications or illicit drugs, and did not smoke. Specifically, participants were screened at their first visit to ensure they were non-diabetic according to an oral glucose tolerance test (OGTT) [World Health Organization (WHO) 2006 criteria^18^], and had a normal physical examination and routine blood analyses. The protocol was approved by the Alfred Hospital and Monash University Ethics Committees and complied with the Declaration of Helsinki 2004. All subjects provided written informed consent prior to participation.

Participants were characterized for anthropometric measures, body composition, glucose tolerance, insulin sensitivity, blood pressure, white blood cell count, and glomerular filtration rate assessed as estimated GFR (eGFR).

### Research design and measurements

All subjects underwent screening, which involved a medical history, physical examination including blood pressure measurement, and basic laboratory tests including fasting plasma lipid levels, liver function tests, urea, creatinine, eGFR, and electrolytes. Those who were found healthy underwent anthropometric assessments, an OGTT, and a hyperinsulinaemic euglycaemic clamp. Prior to metabolic testing, participants were asked to abstain from strenuous exercise and caffeine for at least 24 hours. All metabolic testing was performed after a 12-h overnight fast.

### Anthropometric measurements

Body weight and waist and hip circumferences were measured and waist-to-hip ratio (WHR = waist (cm)/hip (cm)) was calculated as an index of body fat distribution. Body composition was assessed by total body dual-energy X-ray absorptiometry (DEXA) (Lunar Radiation Corp., Madison, WI, USA). Body mass index (BMI) was calculated as [weight (kg)/height^2^ (m^2^)]. BMI categories were defined as lean = BMI < 25 kg/m^2^, overweight = BMI of 25–29.99 kg/m^2^, obese = BMI ≥ 30 kg/m^2^.

### Metabolic studies

Average systolic and diastolic blood pressure (SBP, DBP) was derived for each participant from three measurements using an automated sphygmomanometer (M6 Automatic BP monitor, Omron, Japan) after at least 5 minutes rest. Mean arterial pressure was calculated as 2 × DBP + SBP/3. A two-hour 75-g OGTT was performed after a 12-h overnight fast and glucose tolerance status was determined by WHO criteria^18^. Plasma glucose concentrations were determined by the glucose oxidase method (ELM 105 Radiometer and YSI 2300 Stat Glucose Analyser).

Insulin sensitivity was measured during a hyperinsulinemic-euglycemic clamp, performed as per our published protocol^19^. In brief, after an overnight fast, we collected baseline blood and tested plasma glucose levels at 0 minute, after which the clamp was initiated by an intravenous bolus injection of insulin (9 mU/kg). A continuous intravenous insulin infusion was then administered for at least 120 minutes at a constant rate of 40 mU per m^2^ body surface area per minute. Plasma glucose was measured every 5 minutes during the clamp and the variable infusion rate of glucose was adjusted to maintain blood glucose at a constant value of about 5 mmol/L. This infusion achieved steady state plasma insulin concentrations and the rate of total insulin-stimulated glucose disposal (M-value) was calculated for the last 30 min of the insulin infusion. We defined insulin resistance as M < 4.7 mg/kg/min^20^. Blood samples collected at baseline were used for analysis of WBC and creatinine. Glomerular filtration rate was estimated by use of the CKD-Epidemiology Collaboration (CKD-EPI) equation^16^.

### Statistical analysis

Statistical analyses were conducted using STATA (version 14). Participant characteristics in total, and according to gender, are presented as means ± standard deviation (SD), or medians [interquartile range], with differences analysed by independent Student’s *t*-tests for continuous and normally distributed variables, and Mann-Whitney U tests for non-normally distributed variables. Normality of distribution was tested using the Shapiro-Wilk test and inspection of frequency histograms. Chi-square test was used to investigate if a significant difference existed in the distribution of BMI categories (lean, overweight, obese) between genders. Pearson correlation (r) was used as a measure of the strength of correlations between each variable and eGFR. Multivariable linear regression analysis was used to assess the association between eGFR as the dependent variable with IS (M) as an independent variable, and to determine the effect of other covariates. We initially plotted the relationship between eGFR and IS and confirmed a linear trend. For multivariable regression models, we adjusted for variables which were significantly correlated to eGFR based on Pearson correlation and those known to associate with eGFR based on previous studies including systolic blood pressure. A two-tailed p-value < 0.05 was considered statistically significant. One-way ANOVA followed by post hoc Tukey test was applied to examine the mean eGFR across BMI categories among the insulin sensitive individuals, and also between insulin sensitive and insulin resistant obese individuals. We also examined the difference in mean eGFR across tertiles of IS and 2-hour glucose post OGTT using one-way ANOVA. For IS, tertile 1 was defined as M between 1.24 and 5.69 mg/kg/min (mean ± SD: 4.05 ± 1.0, N = 35), tertile 2 as M between 5.86 and 8.83 mg/kg/min (7.5 ± 0.9, N = 35) and tertile 3 as M between 8.84 to 17.41 mg/kg/min (11.8 ± 2.7, N = 34). For 2-hour glucose post OGTT, tertile 1 was defined as glucose level between 2.5 and 4.6 mmol/L (mean ± SD: 3.9 ± 0.5, N = 35), tertile 2 as glucose level between 4.6 and 5.8 mmol/L (5.2 ± 0.4, N = 35) and tertile 3 as glucose level between 5.9 and 10.6 mmol/L (7.0 ± 1.2, N = 34). We conducted trend analyses to further investigate the relationships between eGFR and IS, and 2-hour glucose post OGTT. Post hoc, we tested for interactions running the Wald test in the adjusted model and performed secondary multivariable analyses where we found a significant interaction.

## Results

Participant characteristics are presented in Table 1. The majority of the study population (n = 89, 85.6%) were classified overweight or obese according to the WHO BMI classification^21^ (38.5% overweight and 47.1% obese). Twenty-seven participants (26%) were insulin resistant (M < 4.7 mg/kg/min) (Table 2). Males had higher mean systolic blood pressure (SBP), arterial pressure, fasting plasma glucose level, fat free mass (FFM) and serum creatinine level and a higher median waist-to-hip-ratio (WHR) compared to females (Table 1). Females had higher mean percent body fat compared to males. However, there were no significant differences in BMI and IS between genders (Table 1). Males also had a less favorable lipid profile with higher mean total cholesterol and triglycerides, and lower mean high-density lipoprotein cholesterol (HDL-C) levels (Table 1). In univariable analysis, eGFR was negatively related to age (Table 3). Serum creatinine levels were not different across BMI categories (p = 0.4) and between insulin resistant and insulin sensitive groups (p = 0.08).

### Relationship between eGFR and anthropometric measures

**Figure 1:**
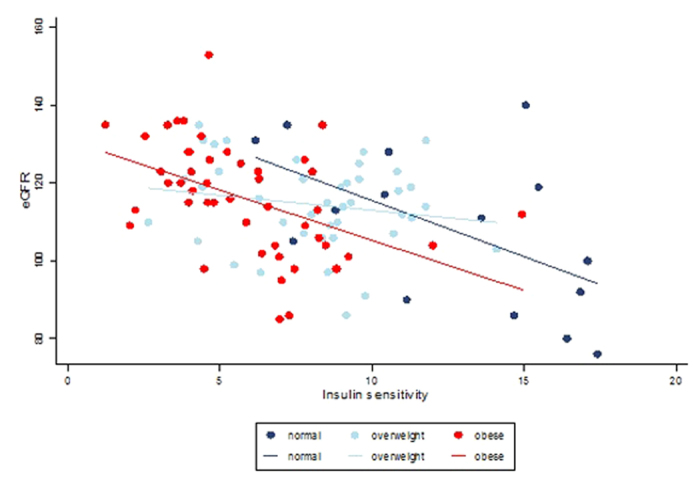
Association of eGFR and insulin sensitivity (M) by BMI.

There was a positive association between eGFR and BMI (r = 0.23, p = 0.02), and waist circumference (r = 0.23, p = 0.02) but eGFR was not associated with percent body fat (r = 0.1, p = 0.3), fat free mass (r = 0.11 p = 0.3) and WHR (r = 0.1, p = 0.3). None of the anthropometric measures were independently related to eGFR after additional adjustment for IS (all p > 0.05). There was a trend for BMI, particularly in the overweight group, interacting on the relationship between eGFR and IS (Fig. 1). However, this interaction did not reach statistical significance (p = 0.07). Using post-hoc Tukey test, when categorized based on IS, there was no significant difference in the mean eGFR between lean, overweight, and obese individuals within insulin sensitive (M ≥ 4.7 mg/kg/min) (p = 0.3) and insulin resistant (M < 4.7 mg/kg/min) groups (p = 0.4).

### Relationship between eGFR, blood pressure, and lipid profile

eGFR was not associated with systolic (r = 0.15, p = 0.1), diastolic (r = 0.14, p = 0.1), or mean arterial blood pressure (r = 0.16, p = 0.1). These associations remained non-significant after adjustment for adiposity (p > 0.1). Regarding lipid profile, eGFR was related to HDL-C (r = −0.2, p = 0.04), but not related to total cholesterol (r = −0.05, p = 0.6), LDL-C (r = −0.03, p = 0.7) and triglyceride levels (r = 0.14, p = 0.2). After adjusting for adiposity, no relationships were found between eGFR and any of the plasma lipid measures (all p > 0.2).

### eGFR and white blood cell count

eGFR was associated with white blood cell count (WBC) (r = 0.21, p = 0.04), but not after adjustment for adiposity (p > 0.1).

### Relationship between eGFR, glycaemic indices and insulin sensitivity

**Figure 2:**
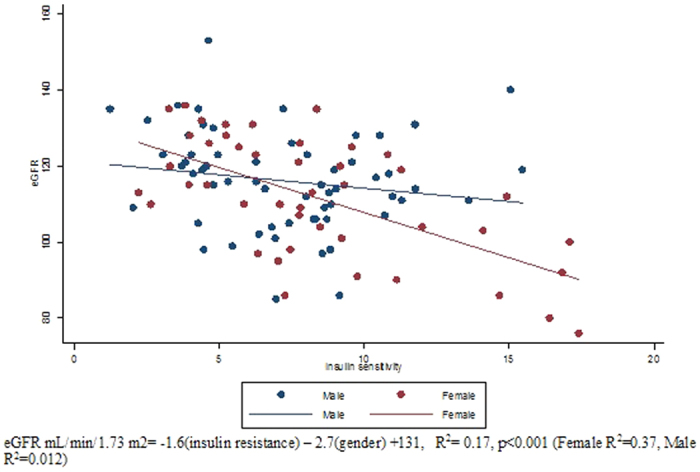
Association of eGFR and insulin sensitivity (M) by gender.

eGFR was positively related to 2-hour glucose post OGTT (r = 0.24, p = 0.01), and inversely related to IS (M) (r = −0.42, p < 0.001) but not related to fasting glucose (r = 0.13, p = 0.2). In addition, we found significant differences in eGFRs across tertiles of IS measured as M (122.94 ± 11.4 mL/min/1.73 m^2^ in the least insulin sensitive group vs 110.26 ± 12.4 mL/min/1.73 m^2^ in the moderately sensitive group vs 109.44 ± 15.3 mL/min/1.73 m^2^ in the most insulin sensitive group; p < 0.0001). Similarly, there were significant differences in eGFRs across tertiles of 2-hour glucose post OGTT (120.74 ± 13.3 mL/min/1.73 m^2^ in the first tertile with the highest 2-hour glucose vs 111.69 ± 14.8 mL/min/1.73 m^2^ in the second tertile vs 110.54 ± 13.2 mL/min/1.73 m^2^ in the third tertile with the lowest 2-hour glucose). In the multivariable analysis, the relationship between eGFR and IS remained significant after additional adjustments for, BMI, SBP, and WBC (Table 3). Similarly, the relationship between eGFR and 2-hour glucose post OGTT remained significant after additional adjustments for BMI, SBP, and WBC (p < 0.01). When both IS and 2-hour glucose post OGTT were in the model adjusted for BMI and SBP, IS was the only significant predictor of eGFR (p = 0.009). Use of waist circumference, WHR, fat free mass, or percent body fat instead of BMI yielded similar results regarding the association between eGFR and IS (p < 0.05). However, further analysis revealed a significant interaction by gender on the relationship between eGFR and IS (p = 0.02). Hence, univariable and multivariable linear regression analyses were conducted separately in males and females (Tables 4 and 5, respectively). We used model 1 to assess the relationship between eGFR and IS (M) adjusting for potential confounders including BMI, SBP and WBC. In model 2, we adjusted for model 1 covariates as well as 2-hour glucose post OGTT. Using post hoc Tukey test, among obese individuals (BMI ≥ 30), there was a significant difference in mean eGFR between insulin sensitive (M ≥ 4.7 mg/kg/min) and insulin resistant (M < 4.7 mg/kg/min) groups (p = 0.0001). When divided by gender, we found a significant relationship between eGFR and IS in females (adjusted R^2^ = 0.38, p = 0.005), but not in males (adjusted R^2^ = 0.29, p = 0.9) (Tables 4 and 5; Fig. 2). Among males, 2-hour glucose post OGTT was related to eGFR in the regression analysis (Table 4). Replacing BMI with percent body fat, fat free mass, waist circumference or WHR in separate regression models by gender did not alter these associations (data not shown).

## Discussion

Our study shows that in a predominantly obese but otherwise healthy population of adults, there is a modest positive relationship between measures of adiposity and eGFR, and a strong negative relationship between IS and eGFR, which is independent of adiposity and SBP. Furthermore, our findings suggest a gender difference exists in the relationship between eGFR and IS. In females, IS was the strongest predictor of eGFR. In men, eGFR was most strongly associated with 2-hour glucose post OGTT, which is a marker of both IS and β cell function^22^.

In our study, measures of adiposity (BMI, waist circumference, and percent body fat) were positively related to eGFR after adjustment for age and gender. However, after additional adjustment for IS, IS but not adiposity was related to eGFR. Aligned with this, we showed no significant difference in mean eGFR across BMI categories when participants were classified by M-value as insulin sensitive or resistant. This suggests that insulin resistance, rather than obesity, may be the key driver in the development of glomerular hyperfiltration, which has been shown to be an early predictor of albuminuria, chronic renal disease and renal failure^4^,^5^,^23^. Several studies have reported an association between measures of adiposity, and glomerular hyperfiltration. A progressive increase in GFR has been observed with increasing BMI even in non-obese healthy individuals^9^. BMI has been shown to directly affect renal haemodynamics and alter the afferent/efferent balance, which could result in glomerular hypertension and hyperfiltration and ultimately, renal injury^9^,^23^. However, the relationship between eGFR and BMI is often not adjusted for insulin resistance^9^,^24^,^25^,^26^. Furthermore, obesity is associated with hypertension, insulin resistance, and dyslipidaemia, all of which are known risk factors for renal disease and it is difficult to differentiate the independent contribution of each of these factors to renal hyperfiltration. There are other studies, however, which have reported reduced GFR and reduced renal plasma flow associated with increased BMI, and especially with central obesity^27^. A greater WHR and increased intra-abdominal pressure due to visceral fat in obese individuals have been found to be associated with renal vein compression, and diminished venous outflow from the renal veins due to increased inferior vena cava pressure^6^,^8^. These could result in slower renal blood flow and decreased glomerular filtration rate.

Our results are consistent with another cross-sectional study of 1,572 young, healthy men which showed that insulin resistance based on homeostatic model assessment (HOMA-IR) was higher in men with hyperfiltration compared to those without hyperfiltration^3^. GFR in this study was calculated using the Cockcroft–Gault equation. Other studies described either no relationship or a positive relationship between GFR and insulin sensitivity. A study of 574 non-diabetic individuals found no relationship between CKD-EPI-calculated eGFR and insulin resistance quantified by an insulin suppression test^28^. The study population was older compared to our study (mean age of 50 years among those without metabolic syndrome and 53 years among those with metabolic syndrome, compared to 31.2 years in our study), with expectedly lower mean eGFR compared to our study sample (89.3 mL/min/1.73 m^2^ among those without metabolic syndrome and 87.5 mL/min/1.73 m^2^ among those with metabolic syndrome vs 114.3 mL/min/1.73 m^2^ in our study). Another study of 864 non-diabetic participants, calculated GFR based on the Modification of Diet in Renal Disease (MDRD) equation, and IS by the frequently sampled intravenous glucose tolerance test using mathematical modeling methods to measure IS index^29^. This study also found no association between IS index and GFR. Apart from the limitations of GFR based on MDRD^16^, the studied population were again much older than ours (mean age 52.6 ± 1.0). Notably, some of the participants in both studies were on antihypertensive medications (angiotensin-converting-enzyme inhibitors (ACEI) or angiotensin receptor blockers) or statins, which could have confounded their results^28^,^29^. Among previous cross-sectional studies that have reported a positive association between IS and GFR, only one used the hyperinsulinaemic euglycaemic clamp technique to measure IS^7^. This study included exclusively elderly men (mean age 71 ± 0.59 years). As expected, even in the subgroup with normal renal function, the mean GFR was much lower than the mean GFR in our study (65.3 ± 10.4 vs 114.3 ± 14.4 ml/min/1.73 m^2^) and the majority had hypertension (>66%), dyslipidaemia (>85%), or both, with some requiring medications. Therefore, these results are not generalizable to young and/or healthy populations^7^. In young, healthy cohorts as in our study, renal changes initiated by insulin resistance are more likely to manifest as increased GFR, whereas in older individuals with features of metabolic syndrome, the effect of insulin resistance on kidney function is likely to result in decreased GFR. In addition, antihypertensive therapy is an important confounder in the above study owing to its protective effects on kidneys. Other cross-sectional studies that showed a positive association between IS and GFR used surrogate markers, including fasting insulin and HOMA-IR to evaluate IS and involved older populations with lower mean GFR or established renal disease, with comorbidities such as hypertension and dyslipidaemia, and, in one study, with diabetes^30^,^31^,^32^,^33^.

The pathophysiological mechanisms underlying our observations are likely complex and we suggest that these are primarily associated with hyperinsulinaemia secondary to insulin resistance. Hyperinsulinaemia has been shown in animal and human experimental studies to induce renal afferent vasodilatation, increase fractional sodium reabsorption, activate the renin-angiotensin system, and increase GFR^34^,^35^,^36^,^37^,^38^. Insulin also promotes the production of insulin-like growth factor that has been shown to increase renal plasma flow and GFR^39^,^40^. Moreover, impaired IS and compensatory hyperinsulinaemia is associated with increased sympathetic nervous system activity, increased adipocytokines such as IL-6, TNF-α, leptin, and resistin leading to increased chronic low-grade inflammation and endothelial dysfunction^8^,^41^. A combination of these factors can lead to altered renal haemodynamics that may eventually lead to glomerular hyperfiltration. Glomerular hyperfiltration can then increase vascular permeability resulting in albuminuria^41^. Notably, glomerular hyperfiltration has been shown to improve after interventions such as sodium restriction and weight reduction^42^,^43^. This may have important clinical implications as appropriate screening and implementing early interventions to improve IS may reverse hyperfiltration and prevent progression to albuminuria and further renal injury, particularly in those at high risk of impaired IS who may not necessarily be overweight or obese.

To our knowledge, no previous study has investigated gender differences in metabolic predictors of eGFR in relatively young non-diabetic adults. Sex hormones and different patterns of fat distribution between males and females may play a role in the development of glomerular hyperfiltration and likely interact with other factors affecting renal function. Gender differences in renal haemodynamic function, renal response to glycaemia and ACEIs, and patterns of microalbuminuria have been reported in animal and human studies^12^,^13^,^44^. Men have been reported to have a higher risk of developing chronic kidney disease and a faster progression to end-stage renal disease^14^,^45^. Our data suggests that insulin resistance may increase glomerular hyperfiltration in females, whereas in males, hyperfiltration develops only after glucose intolerance occurs and perhaps contributes to a more rapid decline in renal function with the further deterioration of glucose tolerance. This hypothesis needs to be tested in prospective and intervention studies.

Our small sample size and the predominance of overweight and obese individuals in our study sample limits the generalizability of our findings. Additionally, directionality and causality of the observed relationships cannot be ascertained in these cross-sectional analyses. We did not use inulin clearance measurement, which is considered the gold-standard method of determining renal function^46^. Calculated GFR may also underestimate true GFR in normal and high-to-normal ranges, especially in obese populations, although the CKD-EPI formula appears to be more accurate than the other commonly used equation- MDRD^16^. Moreover, not monitoring sodium and protein intake in participants’ diets and the absence of urinary measures of renal function including 24-hour urinary sodium, does not allow for comprehensive assessment of other factors that might have affected eGFR in our study. Further studies investigating renal hemodynamics such as intra-renal sodium loading, renin-angiotensin system activation, and tubular glomerular feedback, in addition to renal injury markers, such as kidney injury molecule-1, liver fatty acid binding protein, and interleukin-18^47^, would add to our understanding of the mechanisms of hyperfiltration in the context of insulin resistance. Finally, we did not find any association between eGFR and SBP. This is likely due to the narrow range of blood pressure in our population as all participants had normal blood pressure levels. However, to the best of our knowledge, this is the first study examining the relationship between eGFR, adiposity, and IS based on the hyperinsulinaemic euglycaemic clamp in a predominantly obese but otherwise healthy cohort and investigating gender differences in predictors of eGFR. We were also able to adjust for adiposity based on percent body fat from DEXA which, to our knowledge, has not been previously reported.

We advance the knowledge in this field by demonstrating that higher eGFR was more closely related to insulin resistance than to obesity. In addition, we show gender differences in predictors of eGFR. Gender-specific analyses revealed IS as the main predictor of eGFR in women; whereas 2-hour glucose post OGTT was particularly important in men. Our findings suggest that impaired IS, via increasing glomerular filtration rate and potentially hyperfiltration, may play an independent role in the pathophysiology of renal injury, particularly in females. Given the limitations of this cross-sectional study, randomised clinical trials are needed to establish causality and to examine the effect of improving insulin sensitivity in reversing glomerular hyperfiltration and preventing renal impairment.

## Additional Information

**How to cite this article**: Naderpoor, N. *et al*. Higher glomerular filtration rate is related to insulin resistance but not to obesity in a predominantly obese non-diabetic cohort. *Sci. Rep.* **7**, 45522; doi: 10.1038/srep45522 (2017).

**Publisher's note:** Springer Nature remains neutral with regard to jurisdictional claims in published maps and institutional affiliations.

## Figures and Tables

**Table 1:** Anthropometric and metabolic profile.

Variable	All participants	Male (n = 60, 57.7%)	Female (n = 44, 42.3%)	P
*Age (year)	29.00 [14]	29.00 [14]	28.00 [18]	0.8
**Anthropometric**				
*BMI (Kg/m^2^)	29.56 [6.1]	29.14 [7.6]	30.15 [5.1]	0.6
Percent body fat	36.09 ± 10.4	31.18 ± 9.0	42.96 ± 8.1	<0.001
Fat free mass (kg)	54.64 ± 12.7	62.27 ± 10.6	43.98 ± 5.7	<0.001
*Waist-to-hip ratio	0.91 [0.1]	0.96 [0.1]	0.89 [0.1]	<0.001
*Waist circumference (cm)	99.25 [17]	100.75 [16]	96.50 [17]	0.08
**Metabolic**				
Fasting plasma glucose level (mmol/L)	4.64 ± 0.5	4.76 ± 0.5	4.49 ± 0.5	0.01
2-hour glucose postOGTT (mmol/L)	5.36 ± 1.5	5.20 ± 1.4	5.56 ± 1.6	0.2
M (mg/kg/min)	7.74 ± 3.6	7.31 ± 3.1	8.33 ± 4.1	0.2
Creatinine (μmol/L)	68.58 ± 14.0	75.68 ± 10.73	58.19 ± 11.7	<0.001
eGFR (mL/min/1.73 m^2^)	114.26 ± 14.4	116.12 ± 13.1	111.73 ± 15.8	0.1
Systolic blood pressure (mmHg)	119.99 ± 12.6	123.45 ± 12.5	115.28 ± 11.2	0.001
Diastolic blood pressure (mmHg)	77.46 ± 9.5	78.14 ± 9.8	76.53 ± 9.2	0.3
Mean arterial pressure (mmHg)	91.64 ± 9.6	93.24 ± 9.8	89.45 ± 9.0	0.04
Total cholesterol (mmol/L)	4.64 ± 0.8	4.79 ± 0.9	4.45 ± 0.7	0.03
*Triglyceride (mmol/L)	1.14 [0.8]	1.40 [1.2]	1.00 [0.6]	<0.001
HDL-C (mmol/L)	1.22 ± 0.3	1.15 ± 0.3	1.31 ± 0.3	0.008
LDL-C (mmol/L)	2.84 ± 0.7	2.93 ± 0.8	2.70 ± 0.6	0.1
**Inflammatory marker**				
*White blood cell count (x10^9^/L)	6.05 [2.1]	6.10 [2.3]	6.00 [2.0]	0.9

*Not normally distributed variable.

Data presented as mean ± standard deviation for normally distributed variables and median [interquartile range] for not normally distributed variables.

**Table 2:** Insulin sensitivity based on BMI category.

BMI category	Insulin sensitive (M ≥ 4.7 mg/kg/min)	Insulin resistant (M < 4.7 mg/kg/min)	Total
Lean (BMI < 25)	15	0	15
Overweight (BMI 25 to 29.99)	35	6	41
Obese (BMI ≥ 30)	27	21	48
Total	77	27	104

**Table 3:** Multiple regression analysis of eGFR (dependent variable) and insulin sensitivity (independent variable) after adjustment for covariates.

Variable	Univariable		Multivariable	
	β (95% CI)	p	β (95% CI)	p
BMI	0.6 (0.1 to 1.1)	0.02	−0.1 (−0.7 to 0.5)	0.7
SBP	0.2 (−0.1 to 0.4)	0.1	0.1 (−0.06 to 0.3)	0.2
WBC	1.70 (0.1 to 3.3)	0.04	0.5 (−1.1 to 2.1)	0.6
Insulin Sensitivity (M)	−1.7 (−2.4 to −1.0)	<0.001	−1.5 (−2.5 to −0.6)	0.002
2-hour glucose post OGTT	0.2 (0.5 to 4.1)	0.01	0.9 (−1.0 to 2.7)	0.4

eGFR: estimated glomerular filtration rate; BMI: Body mass index; β: unstandardized coefficient, CI: confidence interval, M: insulin sensitivity; p: p-value; SBP: systolic blood pressure; WBC: white blood cell count.

**Table 4:** Multiple regression analysis of eGFR and insulin sensitivity in males.

Variable			Multivariable			
	Univariable		Model 1		Model 2	
	β (95% CI)	p	β (95% CI)	p	β (95% CI)	p
BMI	0.1 (−0.6 to 0.7)	0.8	−0.4 (−1.2 to 0.5)	0.4	−0.3 (−1.1 to 0.5)	0.5
SBP	0.1 (−0.1 to 0.4)	0.3	0.2 (−0.1 to 0.4)	0.3	0.2 (−0.1 to 0.4)	0.3
WBC	0.7 (−1.3 to 2.7)	0.5	0.2 (−1.9 to 2.4)	0.8	−0.5 (−2.7 to 1.6)	0.6
Insulin sensitivity (M)	−0.7 (−1.8 to 0.4)	0.2	−0.9 (−2.4 to 0.5)	0.2	−0.2 (−1.7 to 1.3)	0.8
2-hour glucose post OGTT	3.4 (1.2 to 5.7)	0.004			3.6 (0.8 to 6.4)	0.01

Model 1: Adjusted for BMI, SBP, WBC, insulin sensitivity(M).

Model 2: Adjusted for BMI, SBP, WBC, insulin sensitivity(M), 2-hour glucose post OGTT.

**Table 5:** Multiple regression analysis of eGFR and insulin sensitivity in females.

Variable			Multivariable			
	Univariable		Model 1		Model 2	
	β (95% CI)	p	β (95% CI)	p	β (95% CI)	p
ΒΜΙ	1.3 (0.5 το 2.1)	0.003	0.4 (−0.4 tο 1.3)	0.3	0.4 (−0.4 tο 1.3)	0.3
SBP	0.1 (−0.4 το 0.5)	0.7	0.1 (−0.2 tο 0.5)	0.3	0.1 (−0.3 tο 0.5)	0.5
WBC	3.0 (0.5 to 5.6)	0.02	1.3 (−1.1 to 3.6)	0.3	1.2 (−1.2 to 3.6)	0.3
Insulin sensitivity (M)	−2.4 (−3.3 to −1.4)	0.000	−1.8 (−3.1 to −0.6)	0.003	−1.9 (−3.2 to −0.6)	0.005
2-hour glucose post OGTT	1.5 (−1.5 to 4.6)	0.3			−0.3 (−2.9 to 2.3)	0.8

Model 1: Adjusted for BMI, SBP, WBC, insulin sensitivity(M).

Model 2: Adjusted for BMI, SBP, WBC, insulin sensitivity(M), 2-hour glucose post OGTT.
