# Erratum: Higher glomerular filtration rate is related to insulin resistance but not to obesity in a predominantly obese non-diabetic cohort

**DOI:** 10.1038/srep46962

**Published:** 2018-03-19

**Authors:** Negar Naderpoor, Jasmine G. Lyons, Aya Mousa, Sanjeeva Ranasinha, Maximilian P. J. de Courten, Georgia Soldatos, Barbora de Courten

Correction to: Scientific Reports 7: Article number: 4552210.1038/srep45522; published online: 04 03 2017; updated: 03 19 2018

In the original version of this Article, the authors Maximilian P. J. de Courten and Barbora de Courten were incorrectly indexed. These errors have now been corrected.

